# Accurate face alignment and adaptive patch selection for heart rate estimation from videos under realistic scenarios

**DOI:** 10.1371/journal.pone.0197275

**Published:** 2018-05-11

**Authors:** Zhiwei Wang, Xin Yang, Kwang-Ting Cheng

**Affiliations:** 1 School of Electronic Information and Communications, Huazhong University of Science and Technology, Wuhan, Hubei, China; 2 Department of Electronic and Computer Engineering, Hong Kong University of Science and Technology, Hong Kong, China; City University London, UNITED KINGDOM

## Abstract

Non-contact heart rate (HR) measurement from facial videos has attracted high interests due to its convenience and cost effectiveness. However, accurate and robust HR estimation under various realistic scenarios remain a very challenging problem. In this paper, we develop a novel system which can achieve a robust and accurate HR estimation under those challenging scenarios. First, to minimize tracking-artifacts arising from large head motions and facial expressions, we propose a joint face detection and alignment method which can produce alignment-friendly facial bounding boxes with reliable initial facial shapes, facilitating accurate and robust face alignment even in the presence of large pose variations and expressions. Second, different from most existing methods [1–5] which derive pulse signals from predetermined grid cells (i.e. local patches), our patches are varying-sized triangles generated adaptively to exclude negative effects from non-rigid facial motions. Third, we propose an adaptive patch selection method to choose patches which contain skin regions and are more likely to contain useful information, followed by an independent component analysis, for an accurate HR estimate. Extensive experiments on both public datasets and our own dataset demonstrated that, comparing with the state-of-the-art methods [1–3], our method reduces the root mean square error (RMSE) by a large margin, ranging from 12% to 63%, and can provide a robust and accurate estimation under various challenging scenarios.

## Introduction

The rapid advances in electronic devices equipped with various multimedia tools such as digital cameras stimulated the explosive growth of multimedia computing with diverse applications to medical service and health monitoring [6–11]. Human heart rate (HR) measurement is one of the vital signs for clinical diagnosis of many cardiovascular diseases, cardio training guidelines, and many other medical and health monitoring applications. Traditional HR measurement which mainly relies on the optical technique [4, 12–15] or the electric technique, e.g. electrocardiogram (ECG), requires users to physically make specific contact with the devices (e.g. place a fingertip in contact with an optical sensor for the optical methods or place electrodes on the skin of chest and/or limbs for the electric methods), thus is not suitable for continuous and long-term monitoring. Recently, emerging methods [3–5, 16–25] which utilize video-based multimedia data to extract pulse signals have attracted lots of interests as they are non-contact and thus facilitate the application of human HR monitoring to a much broader range of scenarios which are infeasible or inconvenient for conventional contact based approaches. For instance: 1) long-term and ubiquitous heart rate monitoring of premature neonates and elderly whose skin is fragile and damageable by traditional sensors; 2) assisting human emotion recognition [26] by correlating the HR of a subject with facial expressions and speech using a single camera in front of the subject; 3) cardio training guidelines which guide an exerciser to achieve the target of fat burning, cardio training, endurance training, etc. and meanwhile to ensure a proper workout intensity by continuously monitoring the exerciser using a sport equipment (e.g., running machine) with a built-in front camera.

The underlying idea behind a facial video-based HR estimation method is that every heart beat pumps the blood into faces, resulting in volumetric changes in facial blood vessels and in turn changes the reflection of the incident ambient light. Several methods have been proposed for video-based HR estimation; however, an accurate HR measurement remains challenging under realistic scenarios: 1) large tracking-artifacts due to uncontrolled head motions resulting in large misalignment-caused intensity changes which are mixed with the pulse-related signal, and 2) extremely low signal strength of the pulse-related waveform due to the tiny amount of blood (only 2% to 5% of the total blood in a body) in the facial skin vascular bed, and a small change (∼5%) of the blood volume in sync with the cardiovascular pulse [5]. The pulse-related signal is embedded in various other noise sources (including motion-induced color changes, dynamic illumination changes in mobile scenarios, etc.) whose strength is several orders of magnitude greater than those of pulse-related color changes.

Several efforts have been made to address these two challenges. To eliminate motion-induced tracking-artifacts, the authors in [3, 4, 19] employed face detection and/or face tracking to localize a bounding box encompassing an entire face on every video frame. All pixels within the bounding box are averaged for the pulse estimation to smooth out small motion changes. However, the bounding box localized by existing face detection/tracking methods could be sensitive to large head motions, illumination and/or background changes, and thus fluctuates from frame to frame. To achieve high accuracy, these methods therefore require the user to keep the head still during the process of HR estimation. Moreover, facial bounding boxes inevitably contain non-skin pixels, such as hair, beards and background, disturbing the true pulse related signals and yielding errors. To address these limitations, recent methods [1, 2, 5] employed state-of-the-art face alignment methods to precisely localize a set of facial landmark points on every video frames and estimate HR from local patches including only skin pixels. For instance, Li *et al.* [2] used the Discriminative Response Map Fitting (DRMF) method [27] to detect facial points on the first frame and then tracked the facial points in the subsequent frames by the Kanade-Lucas-Tomasi (KLT) algorithm [28]. Kumar *et al.* [5] divided an entire facial region into seven sub-regions by the deformable face fitting algorithm [29]. Lam *et al.* [1] located and tracked facial points by a facial landmark fitting tracker [30]. The problem, however, is that existing face alignment methods [31–33] are bounding box dependent and initial shape dependent. Large head motions which do occur in practical scenarios could no longer guarantee a convergence of facial shape model and consequently yield large misalignment errors and HR measurement errors [34]. Therefore, for HR measurement in realistic scenarios, there is a need for developing a highly accurate face alignment method which is robust with respect to various head motions.

To recover subtle pulse signals from a mixture of various sources, Xu *et al.* [4], under the assumption that the environmental lighting remains constant during measurement period, utilized the pixel quotient differences of every two adjacent frames to simulate the pulse-related color changes. But in practice the assumption of constant environmental illumination is usually not valid. To address this problem, Li *et al.* [2] segmented the background region of a video and computed the mean green value of the background in each frame to form a background lighting signal. The pulse signal was obtained by subtracting the background signal from the video-recorded facial signal. Other methods formulated it as a Blind Source Separation (BSS) problem and applied Independent Component Analysis (ICA) to extract the unobserved signal (i.e. the pulse signal) from a set of observations (i.e. intensity signals from the facial video) that are composed of linear mixtures of the underlying sources. For instance, Poh *et al.* [3, 19] averaged pixel values of the red (R), green (G) and blue (B) traces in a facial region separately and utilized the R, G, and B signals as the observations of ICA for pulse signal decomposition. Instead of using all RGB traces as [3, 19], the authors in [1] proposed to utilize only the green channel for extracting the pulse signal based on the fact that the absorption spectra of the hemoglobin and oxyhemoglobin in blood peaks at around 520–580 nm [35], which is in a similar passband range of the green channel [5]. Several pairs of local patches are randomly selected from the face region as observations of ICA. Each patch pair is used to derive a HR hypothesis and the hypothesis with the greatest consensus is reported as the final HR estimate. The performance achieved by [1] on a public dataset is superior to those of [3, 19] and several other state-of-the-art methods. However, random patch pair selection can hardly guarantee a high-quality result, especially when the number of inliers (i.e. patches based on which a true HR can be derived) is small. Similarly, the authors in [5] utilized only the green channel of the signal and improved the signal-to-noise ratio (SNR) by partitioning a face into a set of small patches based on facial landmark points and dynamically assign weights to patches according to how likely a patch can provide an accurate HR estimation. However, the size of a patch significantly affects the HR measurement accuracy [1, 5]. A small patch is more robust to non-rigid facial changes (e.g. facial expressions) but more sensitive to misalignment errors; a large patch is less resilient to non-rigid facial changes while more robust to misalignment. To date, the problem of dynamically determining the size and shape of a patch and adaptively choosing the most reliable patches for HR estimation has not been addressed.

In this paper, we aim at robust and accurate HR estimation under realistic scenarios even when there is a large head motion (e.g. when a user is walking or talking) and when the surrounding illumination changes are significant and highly dynamic (e.g. when a user is watching TV, etc.). First, we propose a joint face detection and alignment method which can robustly provide accurate facial landmarks even when there are large head motions. Instead of detecting face and localizing facial landmarks in separate and independent steps as most existing methods [1, 2, 5] did, our method provide a bounding box with initial facial landmarks which can guarantee fast convergence from an initial estimated shape to the actual shape even in the presence of large head pose variations. Second, we apply the Delaunay Triangulation (DT) method to the localized landmarks to approximate the non-rigid face surface using a set of triangles. Each triangle is then used as a local patch for the subsequent HR estimation. As the shape of each triangle changes with non-rigid facial motion so as to cover the same skin region, thus our local patches are robust to non-rigid motions. Third, we propose an adaptive patch selection method to filter out non-skin patches (e.g. eyes, glasses, beards, etc.) and identify reliable patches which are more likely to be useful for deriving correct pulse signal. That is to say, our method can effectively remove potential noises and in turn improve SNR. More specifically, a color image is first converted to a skin probability map (SPM) via a Naive Bayesian Model, where each pixel represents its likelihood of being a skin pixel. Then we calculate the average of the skin probabilities in a patch to determine whether the patch is a non-skin patch or not by thresholding. Patches whose sizes remain stable along the temporal axis are considered as reliable patches which are then used for ICA analysis for better recovering subtle pulse signals and thus for robust HR estimation. In summary, our key contributions include:

- A novel joint face detection and alignment method which enables robust and accurate face alignment even in the presence of large head motions, minimizing the tracking-artifacts for HR estimation.
- An adaptive local patch selection method which can effectively improve SNR by filtering out non-skin patches and discovering reliable ones for robust HR estimation.

We test our proposed method on 487 challenging videos from the MAHNOB-HCI dataset [36]. This dataset contains facial videos with variant challenges including head pose variations, facial expressions and dynamic illuminations, well simulating the realistic scenarios. We compare our method with the state-of-the-art methods [1–3] on this dataset and the experimental results show that our method significantly outperforms [1–3] by reducing the RMSE by 12% to 63%. In addition, we evaluated the impact of two key components in our method on a self-collected dataset. Experimental results demonstrate that the accurate face alignment improves the overall performance from 9.2 RMSE to 5.2 RMSE and the adaptive patch selection further reduces the RMSE to 2.4.

## Methods

### Problem definition

The facial video records the illumination of light reflected back from a face region *P*(*x*, *y*, *t*), where *x*, *y*, and *t* are two dimensional coordinates of a video frame and a temporal point.

**Fig 1:**
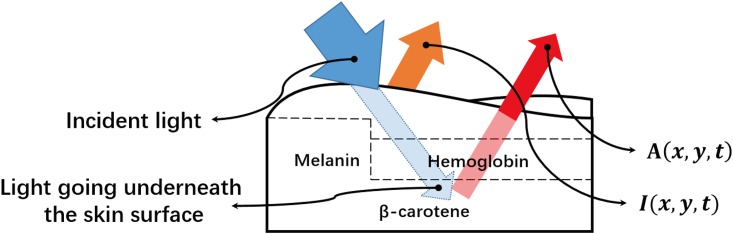
The reflection and absorption of environmental light by human skin.

In general, the reflected light recorded by a video camera is a summation of two parts: (i) *I*(*x*, *y*, *t*)—the light directly reflected by the skin surface which is characterized by the skin’s bidirectional reflectance distribution function (BRDF) [5], as denoted by the orange arrow in Fig 1, and (ii) *A*(*x*, *y*, *t*)—the light travels underneath the skin and then is reflected back after absorbance by various pigments in tissues, including hemoglobin in blood vessels in dermis, melanin in epidermis and *β*-carotene in subcutaneous fat tissue [4], as denoted by the red arrow in Fig 1:

$$\begin{array}{c} P(x,y,t)=I(x,y,t)+A(x,y,t) \end{array}$$ (1)

In Eq (1), *I*(*x*, *y*, *t*) mainly relies on the illumination of the surrounding environments and *A*(*x*, *y*, *t*) relies on both the environmental lightings and the amount of light absorbed by the skin. Generally, the melanin and *β*-carotene remain static in the skin during the period of video recording, yielding a constant light absorbance. Every heart beat changes the volume of blood in facial vessels. As a result, hemoglobin concentration varies in sync with heart beat and in turn synchronously changes the amount of light absorption. Such absorption changes are called the photoplethysmogram (PPG) signal. We define *A*_*h*_(*x*, *y*, *t*) as the light reflected after absorbance by hemoglobin and *A*_*m*_(*x*, *y*, *t*) as the light reflected after absorbance by melanin and *β*-carotene, then *A*(*x*, *y*, *t*) can be represented as the sum of *A*_*h*_(*x*, *y*, *t*) and *A*_*m*_(*x*, *y*, *t*) as shown in Eq (2):

$$\begin{array}{c} A\left(x,y,t\right)=A_{m}\left(x,y,t\right)+A_{h}\left(x,y,t\right) \end{array}$$ (2)

Combining Eqs (1) and (2) we have:

$$\begin{array}{c} P(x,y,t)=\alpha (x,y)p(t)+\beta (x,y)w(t) \end{array}$$ (3)

where *α*(*x*, *y*)*p*(*t*) = *A*_*h*_(*x*, *y*, *t*) and *β*(*x*, *y*)*w*(*t*) = *I*(*x*, *y*, *t*) + *A*_*m*_(*x*, *y*, *t*).

Eq (3) indicates that the intensity of a facial video can be decomposed into two basic signals: the PPG signal related part *p*(*t*) and environmental illumination related part *w*(*t*). *p*(*t*) and *w*(*t*) are weighted by location-dependent factors *α*(*x*, *y*) and *β*(*x*, *y*) respectively. More specifically, *α*(*x*, *y*) and *β*(*x*, *y*) depend on the intensity of incident light, the incident angle of the light and the amount of pigments at different locations over a face. The key task of HR estimation from facial videos is to extract *p*(*t*) from *P*(*x*, *y*, *t*) without prior knowledge about *α*(*x*, *y*), *β*(*x*, *y*) and *w*(*t*).

### Framework of the proposed method

**Fig 2:**
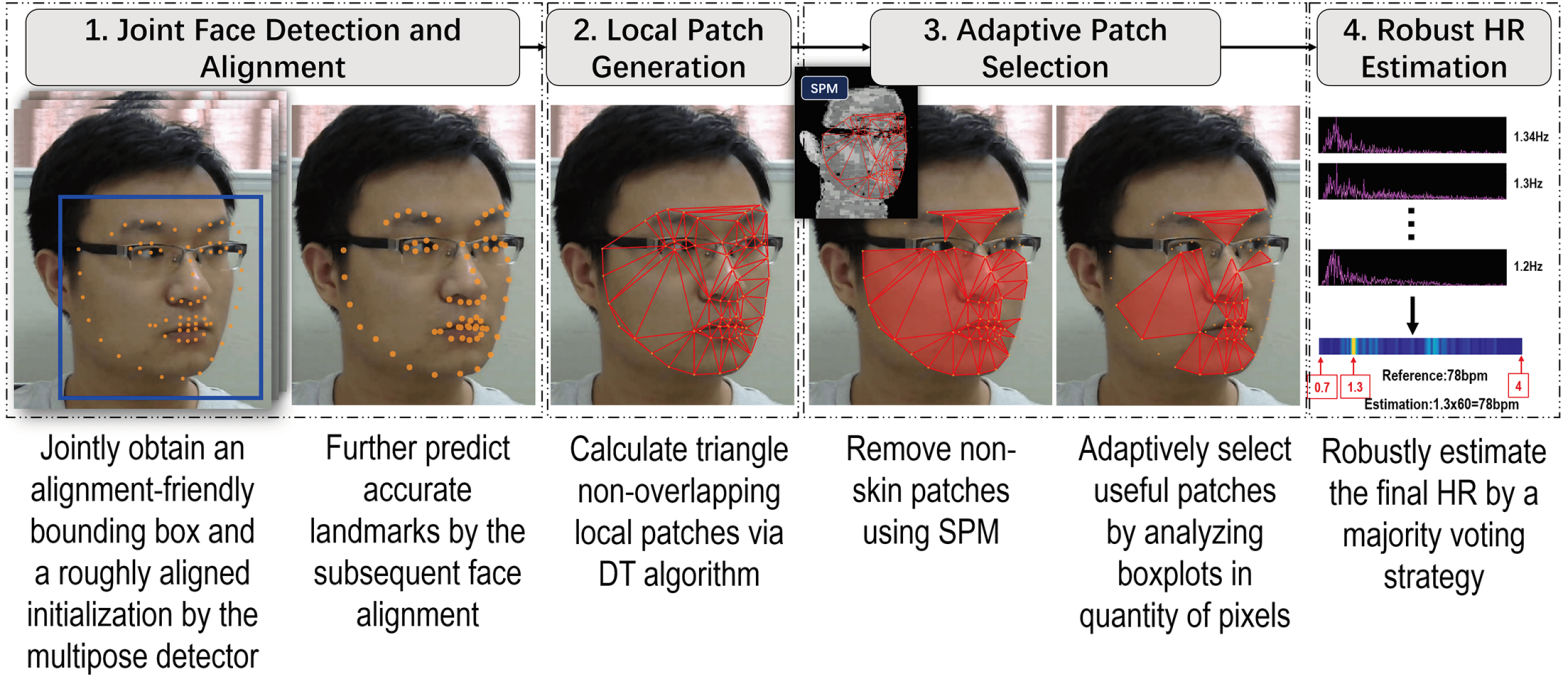
Framework of the proposed method.

Our method consists of four key components to ensure an accurate solution of extracting *p*(*t*) from Eq (3). Fig 2 illustrates the framework of our method.

First, a joint face detection and alignment method is applied to every video frame to localize facial landmarks which is robust to large head poses and facial expressions (Sec. Joint Face Detection and Alignment for Minimizing Tracking-Artifacts). Second, on every video frame we connect facial landmarks via the DT algorithm [37] to form a set of triangles. Each triangle is used as a local patch, corresponding to a unique skin region. After that, we adaptively select a subset of local patches which include only skin pixels and are more likely to achieve an accurate HR (Sec. Local Patch Generation and Adaptive Selection). Finally, the final HR estimation is derived from all adaptive selected local patches via a majority voting strategy (Sec. Robust HR Estimation).

### Joint face detection and alignment for minimizing tracking-artifacts

**Fig 3:**
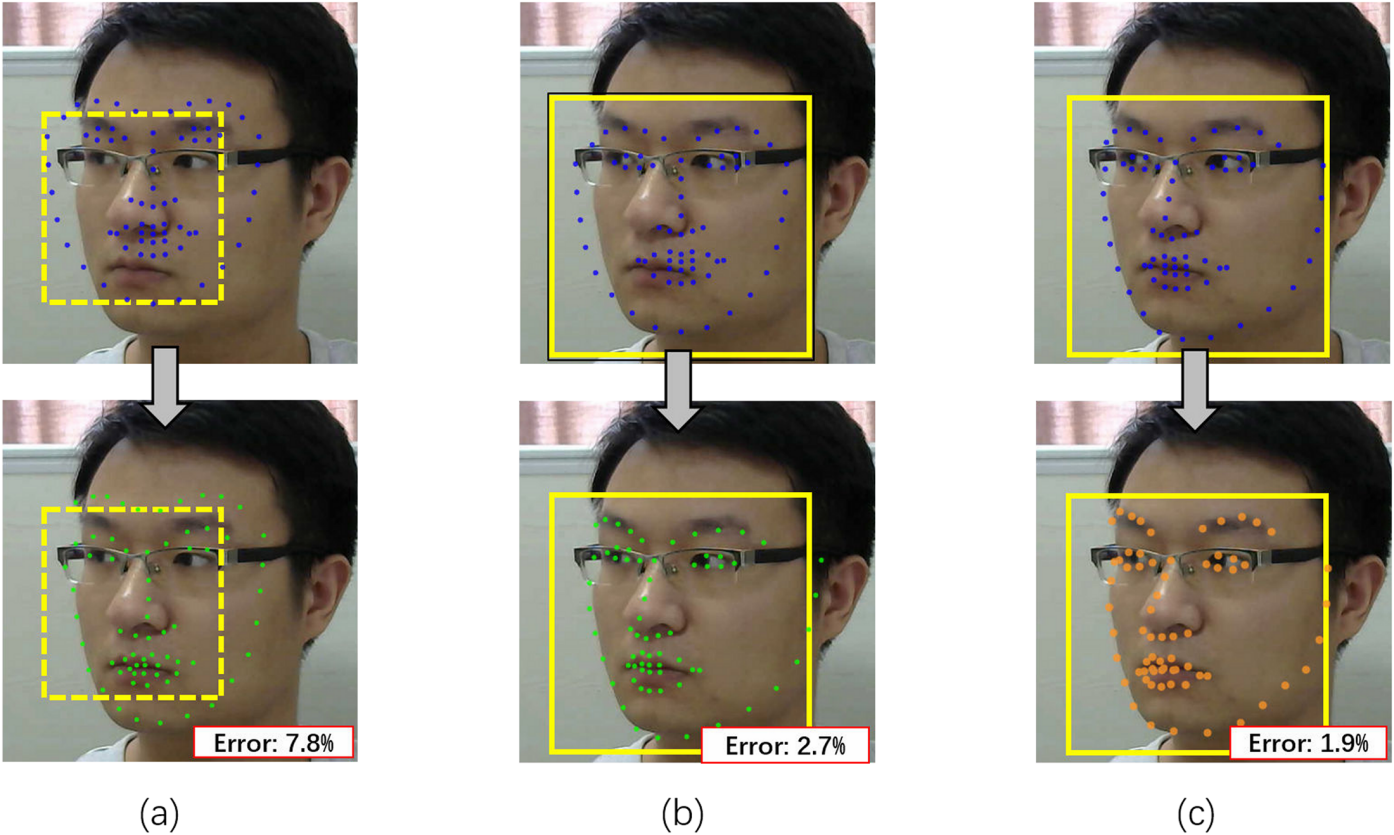
Performance comparison based on different methods for computing the bounding box and the initial shape. (a) When using a bounding box provided by Viola-Jones detector [39] and the mean shape for initialization, the alignment error is large (i.e. 7.8% (averaged point-to-point distance)/(interpupillary distance)) (b) Using the alignment-friendly bounding box provided by our joint method and using the mean shape as the initial shape reduces the alignment error from 7.8% to 2.7%. (c) Further replacing the initialization method based on the mean shape with our joint method can further reduce the error to 1.9%.

Accurately aligning faces in the presence of large head motions remains a very challenging problem, because of face alignment’s reliance on an initial facial shape starting from which a true shape is derived to fit a face. Most existing face alignment methods [31, 32, 38] utilize the mean shape as an initial shape and determine the size and location of the initial shape according to the bounding box. The mean shape is calculated by averaging all shapes from training samples and the bounding box is provided by a face detector. However, when there is a large head pose variation presented in a video frame, the size and shape of the initial shape determined by the bounding box and the mean shape could be quite different from the actual shape (as shown in the top of Fig 3(a)). Large discrepancy between the initial shape and the actual shape could not be completely rectified by subsequent iterations, yielding large alignment errors and motion artifacts in HR estimation.

**Fig 4:**
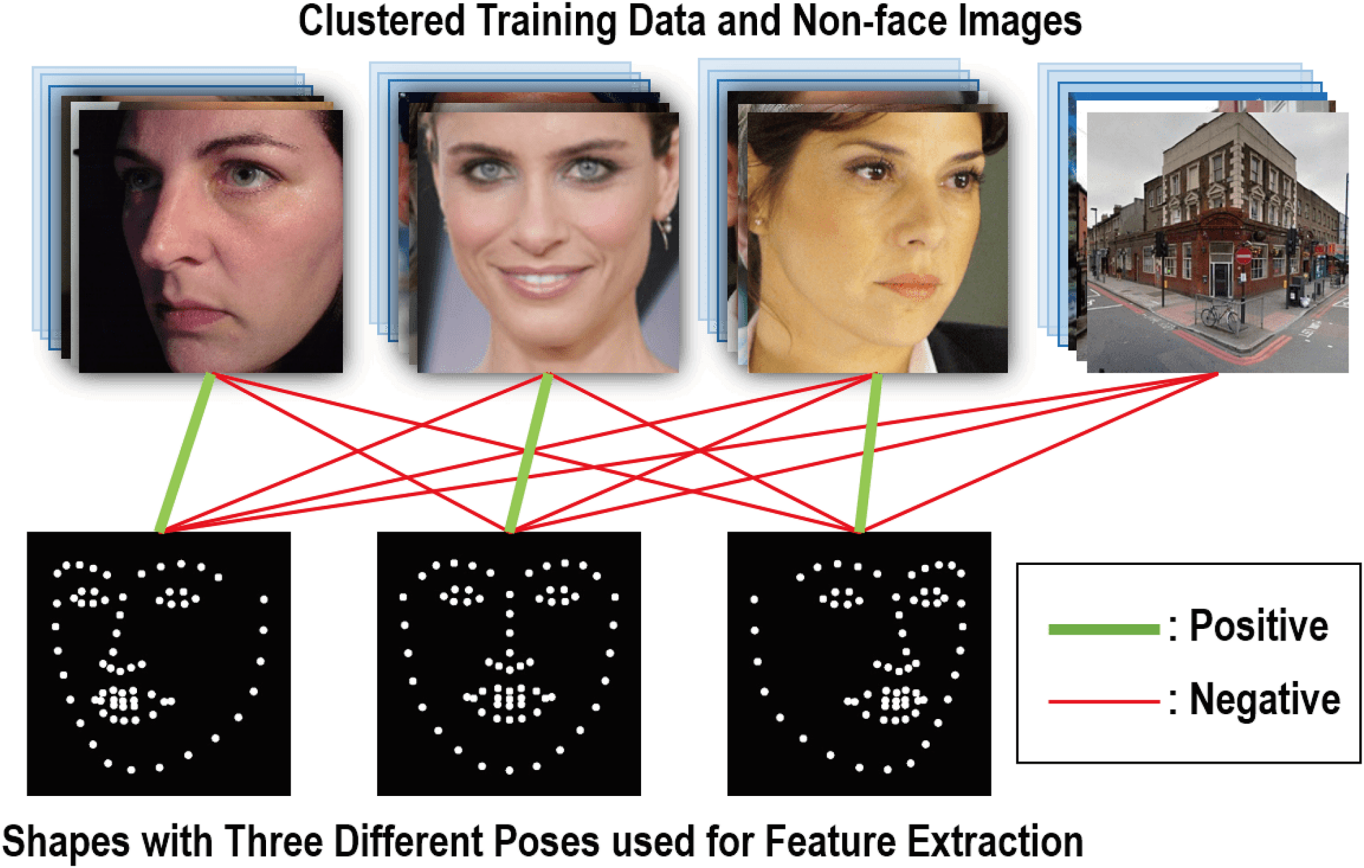
Definition of positive and negative features. Combinations indicated by green lines are used for extracting positive shape-indexed features, and combinations indicated by red lines are used for extracting negative features.

To minimize misalignment errors due to large head motions, we develop a joint face detection and alignment method by combining an alignment-friendly multipose face detector and a reliable initialization of face alignment into a unified cascade framework. Different from conventional face detection method [39] which considers facial regions as positive training samples and non-facial images as negative samples, a positive sample defined in our method is a combination of a facial region and a shape roughly indicating the face pose and a negative sample is otherwise (as shown in Fig 4). Accordingly, the output of our face detector is a combination of a bounding box and a roughly aligned facial shape which acts as a reliable initial shape to ensure a fast and accurate convergence of subsequent face alignment (as shown in Fig 3(c)).

In the training phase, each sample is represented by placing a shape *S* on an image region *x* and then extracting shape-indexed features [40] based on landmarks of *S* from *x*, where *S* = (*l*_1_, ⋯, *l*_*k*_, ⋯, *l*_*K*_) ∈ *R*^2*K*^, *K* is the number of facial points and *l*_*k*_ ∈ *R*^2^ is the 2D coordinates of the *k*-th facial point. Shape-indexed features *ϕ*(*x*, *S*) is calculated by first randomly choosing pairs of pixels in *x*. Then for each pixel in a pixel pair, it is indexed by its location relative to the local coordinates of its closest facial point *l*_*k*_. A shape-indexed pixel pair can be represented in many ways, and in this work we represent a pair using the intensity difference between two pixels for simplicity and efficiency. For positive samples, as its shape *S* is roughly aligned with its actual shape, the corresponding shape-indexed features can well represent facial features. On the other hand, for negative samples, due to large discrepancy between *S* and the actual shape or no face is included in *x*, little representative information for the face can be extracted. Based on these training samples {(*x*, *S*)} and shape-indexed features {*ϕ*(*x*, *S*)}, we construct our face detector using a cascaded random forest as:

$$\begin{array}{c} f^{N}=\sum_{i=1}^{N}C^{i}\left(\phi \left(x,S\right)\right). \end{array}$$ (4)

where each *C*^*i*^ is a weak classifier implemented using a decision tree [41] and *C*^*i*^(*ϕ*(*x*, *S*)) returns a classification score. The decision tree consists of several layers and tree nodes in each layer have two corresponding child nodes. Each tree node selects one shape-indexed feature (i.e. a pixel pair) from a subset of all features in a training sample which can best classify all training samples, and each decision tree weakly splits positives and negatives by a bias threshold. A face detector learns a set of bias thresholds {*θ*^*i*^|*i* = 1, 2, …, *N*} for all decision trees {*C*^*i*^} to acquire the best separation.

In the testing phase, a sliding window is applied to each video frame. Each sliding window *x* goes though weak classifiers sequentially and is rejected immediately whenever *f*^*i*^ < *θ*^*i*^ for any *i* = 1, 2, …, *N*. The combination of (*x*, *S*) which achieves greatest *f*^*N*^ as Eq (5) is considered as the facial bounding box and its corresponding initial facial shape:

$$\begin{array}{c} \underset{S\in \{S_{j}|j=1,2,\ldots ,n\}}{\text{arg}\;\text{max}}\sum_{i=1}^{N}C^{i}\left(\phi \left(x,S\right)\right). \end{array}$$ (5)

**Fig 5:**
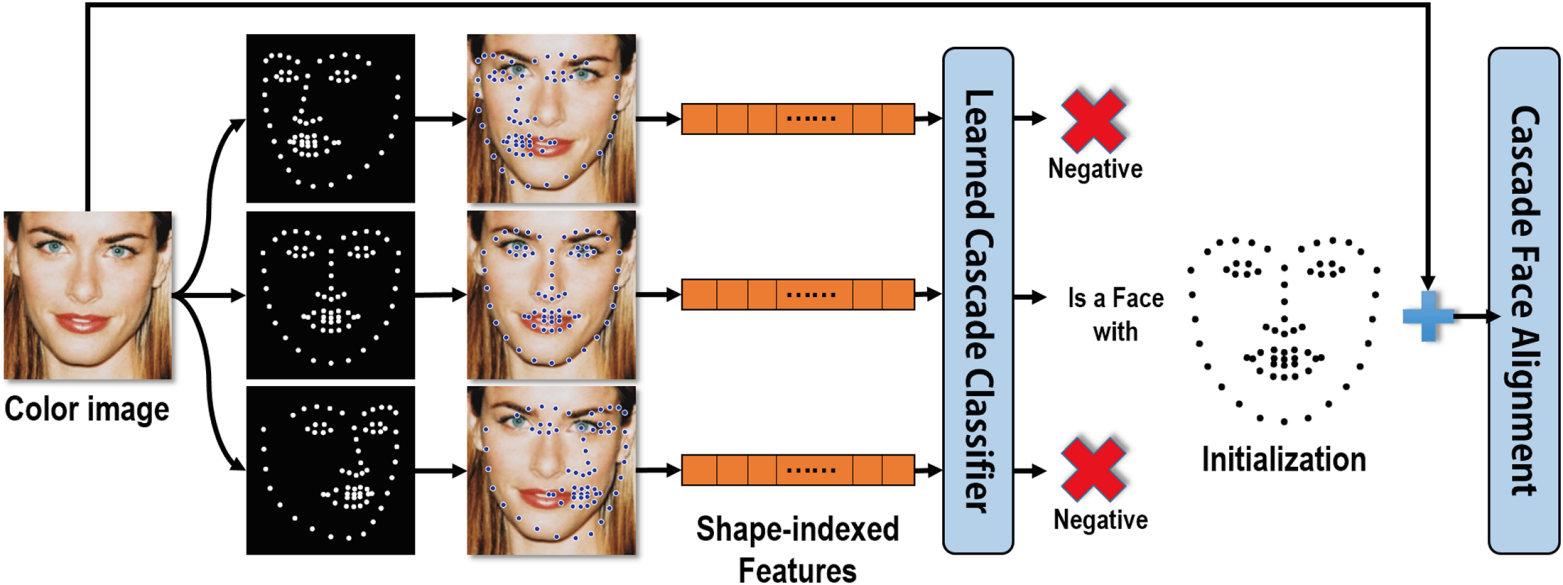
Illustration of testing phase of our joint face detection and alignment.

Afterwards, a face alignment method based on the local binary features (LBF-fast) [38] takes the results (*x*, *S*) from the face detector and performs the alignment as shown in Fig 5. Our detection phase is very fast as most negative image regions are rejected after evaluating only a few weak classifiers and meanwhile can provide an initial shape which can ensure a fast and accurate alignment together with the bounding box.

#### Implementation details

To reduce the time cost for extracting features from a single region *x*, we enumerate a finite number of *S* (i.e. left looking, right looking and frontal shape in our experiment) so as to tolerate a variety of poses when extracting shape-indexed features and meanwhile maintain a reasonable time complexity. The representative shapes for three poses {*S*_*i*_|*i* = 1, 2, 3} are generated by clustering face shapes in trainset based on the Hausdorff distance [42] and obtaining mean shapes from every class as representative shapes {*S*_*i*_}. We extracted 16,866 positive and negative features from 2,811 images and their mirror versions from the HELEN and LFPW datasets provided by [43].

Comparing to the conventional face detection method, our joint method could provide a more alignment-friendly bounding box. By comparing Fig 3(a) and 3(b) our method can reduce the alignment error from 7.8% to 2.7%. Comparing to using mean shape as initialization, utilizing the initial shape generated by our joint method could further reduce the alignment error from 2.7% to 1.9% (as shown in Fig 3(b) and 3(c)). The formal definition of alignment error is presented in Sec. Comparison of face alignment on 300W.

### Local patch generation and adaptive selection

Given the accurately localized facial points, we can generate a local patch by connecting more than three non-repeatable points. To ensure the generated local patches are non-rigid motion resistant and containing meaningful pulse signals, the generated patches need to conform with the following two rules:

- The size of a local patch should be proper, neither too big so as to avoid partial occlusion and be robust to non-rigid motions, nor too small in order to be robust to some misalignment errors.
- Local patches should not overlap each other to avoid redundancy.

We apply the Delaunay Triangulation (DT) method [37] which can well satisfy the above rules to generate local patches. The DT algorithm connects three facial points which are close in distance and meanwhile can maximize the minimum angle of all the angles of the triangle. This requirement can ensure that each generated triangle (i.e. local patch) has a proper size. In addition, the DT algorithm requires that no facial point is inside the circumcircle of any triangle local patch, ensuring that the generated patches are non-overlapped. Exemplar patches generated by DT can be visualized in step 2 of Fig 2.

Two types of local patches could disturb a correct HR estimation: 1) patches containing a majority of non-skin pixels, such as pixels belonging to beards, eyes, glasses, etc., and 2) patches whose sizes constantly change due to head motions. For instance, the size of patches containing eyelids periodically change when eyes are blinking and a patch containing the cheek region close to the nose could be partially occluded by the nose when the head is moving, yielding patch-size variations over time. Those head motion-/facial expression-induced missing pixels result in non-trivial noises when extracting raw signals from patches, and in turn cause inaccuracy for HR estimation, the explanation of which will be further formulated in Sec. Robust HR Estimation.

To eliminate non-skin patches we construct a skin probability map (SPM) based on a RGB video frame. We first collect 78 training images and each of them contains both human skin and background (i.e. non-skin regions). We convert each image from RGB color space to *YCbCr* color space, in which pixels belong to human skins cluster densely even for different races while non-skin pixels spread randomly. We built a two dimensional histogram of *Cb*-*Cr* chromaticity of skin colors, *h*_*skin*_(*Cb*, *Cr*). Each entry in a bin (*Cb*, *Cr*) of the histogram is the number of skin pixels whose color value equals to (*Cb*, *Cr*). Similarly, we built another histogram for the entire set of pixels, *h*_*total*_(*Cb*, *Cr*). By using the Bayes rule, the probability of being a skin for a given (*Cb*, *Cr*) color vector is expressed as:

$$\begin{array}{c} p_{skin}\left(Cb,Cr\right)=\frac{p(Cb,Cr|skin)\times p(skin)}{p(Cb,Cr)} \end{array}$$ (6)

*p*(*Cb*, *Cr*|*skin*) and *p*(*Cb*, *Cr*) are calculated as *h*_*skin*_(*Cb*, *Cr*)/*N*_*skin*_ and *h*_*total*_(*Cb*, *Cr*)/*N*_*total*_ respectively, where *N*_*skin*_ is the number of all skin pixels and *N*_*total*_ is the number of all pixels including skin and non-skin. And *p*(*skin*) is approximated by the fraction of observed skin-like pixels as *p*(*skin*) ≅ *N*_*skin*_/*N*_*total*_. We generate a SPM according to Eq (6) for each video frame. An average of the skin probabilities of each patch is then calculated based on SPM, and those patches with an average skin probability smaller than a predefined threshold (i.e. 0.7 in our experiment) is filtered out as non-skin patches.

Secondly, we select patches whose sizes are relatively stable across all frames in a video. Given a local patch whose pixel counts in a series of *K* video frames are denoted as **N** = (*N*_1_, *N*_2_, …, *N*_*i*_, …, *N*_*K*_), where *N*_*i*_ is the number of its pixels in *i*-th video frame. *N*_*i*_ − *N*_*i*−1_ reflects the pixel count change between two frames, thus we define the pixel change series as **C**:

$$\begin{array}{c} C=(N_{2}-N_{1},N_{3}-N_{2},\ldots ,N_{K}-N_{K-1})\;\;\;\;\;\in Z^{K-1} \end{array}$$ (7)

To normalize every pixel change series into the same scale space, we weight each element in **C** by $K/{\left(\sum_{i=1}^{i=K}N_{i}\right)}^{1/2}$, resulting in:

$$\begin{array}{c} C=\frac{K}{{\left(\sum_{i=1}^{i=K}N_{i}\right)}^{1/2}}(N_{2}-N_{1},N_{3}-N_{2},\ldots ,N_{K}-N_{K-1}) \end{array}$$ (8)

**Fig 6:**
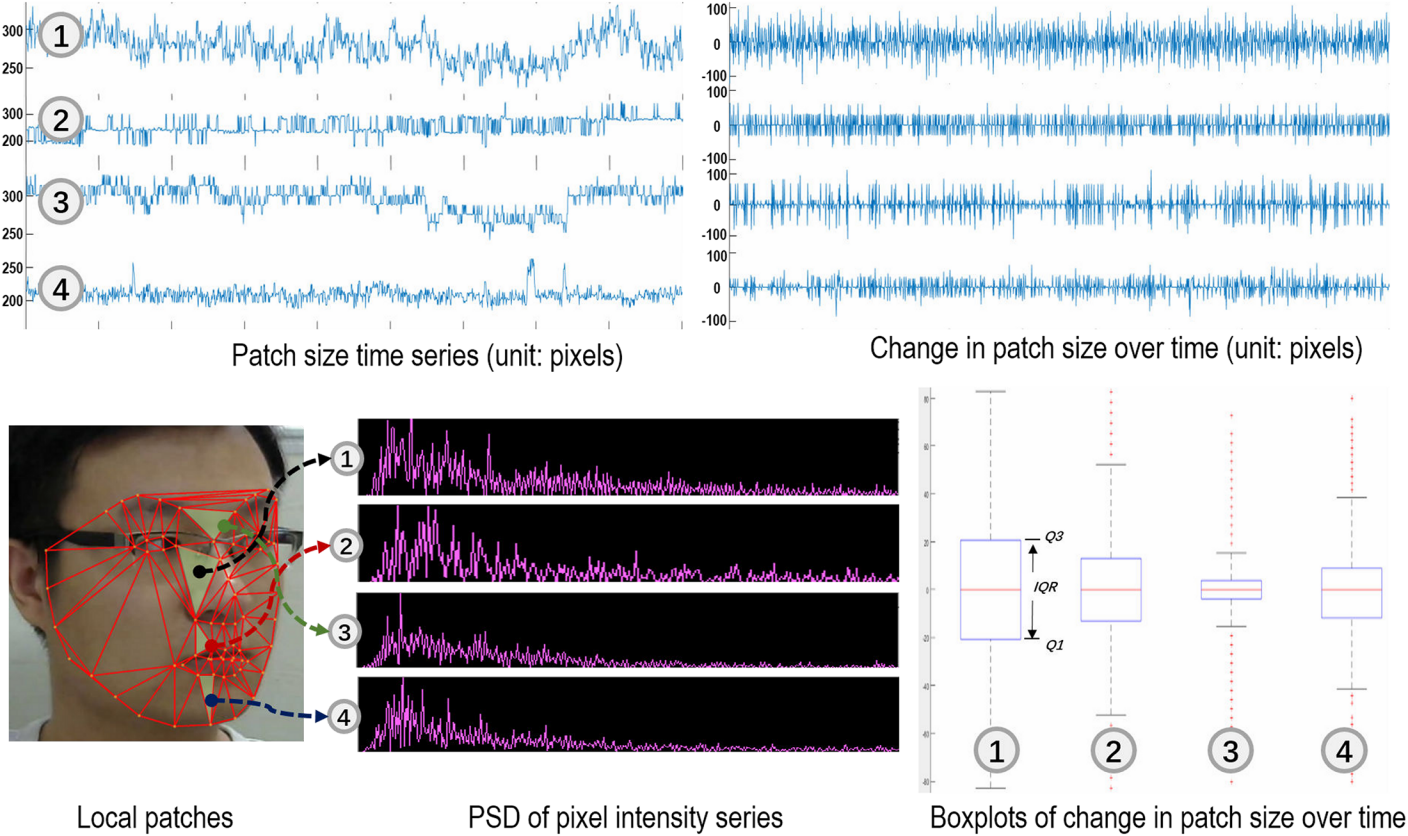
Four local patches are sampled to show their patch size time series (unit: pixels) N, change in patch size over time (unit: pixels) C, boxplots of C and PSD of pixel intensity series. The pixel intensity series is $\bar{P}\left(t\right)$.

Several exemplar patches and their corresponding **N**, **C**, boxplots for **C** and power spectral density (PSD) are shown in Fig 6. The interquartile range (*IQR*), which is calculated as *Q*3 − *Q*1, where *Q*1 and *Q*3 are the first and third quartiles, in boxplots indicates the stability of the patch size along the temporal axis. As observed from Fig 6, the smaller *IQR* is, the more obvious the peak and the less noise the PSD has. For example, the size of the first patch in Fig 6 varies greatly due to the self-occlusion by the nose when there is a large head pose variance. As a result, its corresponding PSD contains multiple peaks and is with many noises, which therefore should not to be used as an observation of ICA for extracting the PPG signal. Therefore, in our algorithm, we calculate *IQR* of each patch, and only select patches with the top 50% smallest *IQR* for estimation of the final HR.

### Robust HR estimation

The final HR estimation is derived from all these selected skin-covered size-stable local patches via a majority voting strategy. Specifically, for each selected local patch, we average all pixels’ green-channel intensity within this patch on every frame to form of its corresponding raw signal observation. Thus, all patches yield a set of observations $\left\{{\bar{P}}_{i}\left(t\right)|i=1,2,\ldots ,n\right\}$, where *n* denotes the total number of adaptively selected local patches. Afterwards, we randomly select a pair of raw signal observations $\left({\bar{P}}_{i}\left(t\right),{\bar{P}}_{j}\left(t\right),i\neq j\right)$ from $\left\{{\bar{P}}_{i}\left(t\right)\right\}$ as [1] did and solve the following equations,

$$\begin{array}{cc} & {\bar{P}}_{i}\left(t\right)={\bar{\alpha }}_{i}p\left(t\right)+{\bar{\beta }}_{i}w\left(t\right)\\ & {\bar{P}}_{j}\left(t\right)={\bar{\alpha }}_{j}p\left(t\right)+{\bar{\beta }}_{j}w\left(t\right) \end{array}$$ (9)

where $\bar{\alpha }=\left[\sum_{x}\sum_{y}\alpha \left(x,y\right)\right]/N$, (*x*, *y*) is the index of a pixel within the local patch whose area is *N*, so is, $\bar{\beta }=\left[\sum_{x}\sum_{y}\beta \left(x,y\right)\right]/N$. Both $\bar{\alpha }$ and $\bar{\beta }$ are time-independent constant.

We generate a *p*(*t*) hypothesis for each patch pair by solving Eq (9) using FastICA [44]. According to the majority voting rule, each *p*(*t*) hypothesis votes a frequency bin which contains the highest peak in the frequency domain ranging from 0.7Hz to 4Hz and the frequency bin receiving the most votes is reported as the final pulse frequency estimate *f*_*HR*_. The final HR estimate from the video is computed as *HR* = 60 × *f*_*HR*_.

## Experimental results

We set up three experiments to evaluate our method. First, we evaluated the performance of face alignment based on our joint method on public datasets with large head pose variations, facial expressions and partial occlusions (Sec. Comparison of face alignment on 300W). Second, we quantified the impact of the two key components of our method—joint detection and alignment and adaptive patch selection using our self-collected dataset, named Pose-Variant HR dataset (Sec. Evaluation on Pose-Variant HR dataset). At last, we assessed the performance of our method on a large and comprehensive dataset MAHNOB-HCI. Specifically, we compared the accuracy of our method for estimating the average HR for a given video clip with several state-of-the-art methods [1–3] (Sec. Comparison of HR Measurement on MAHNOB-HCI). In the following, we briefly describe the datasets and evaluation metrics used in each experiment followed by the corresponding results.

### Comparison of face alignment on 300W

#### Dataset and evaluation metrics

We evaluated the alignment accuracy achieved by our joint method using a challenging dataset *300W* [43]. This dataset includes 3,837 facial images with large scale differences, head pose variations, facial expressions and partial occlusions. We used the same configuration as those in [34, 38, 45] for training. More specifically, we utilized 3,148 training images which consists of 337 images from AFW, 2,000 images from the HELEN training set and 811 images from the LFPW training set. The mirror version of the 3,148 images is also used for training. We evaluated the face alignment accuracy using 689 images, which consists of 330 images from the HELEN test set, 224 images from the LFPW test set and 135 images from iBug. We used the 68 landmark model for face alignment.

The face alignment accuracy is calculated as the normalized alignment error, which is the average Euclidean distance between groundtruth landmarks and the predicted landmarks divided by the inter-ocular distance (measured as the Euclidean distance between the outer corners of the eyes). The average normalized error is used to quantify performance of our joint method. In addition, the Cumulative Error Distribution (CED) curve will be produced, where each value at the *x*-axis is a normalized error and *y*-value of each point indicates the proportion of test images for which the normalized error is less than the *x*-value.

#### Results

**Fig 7:**
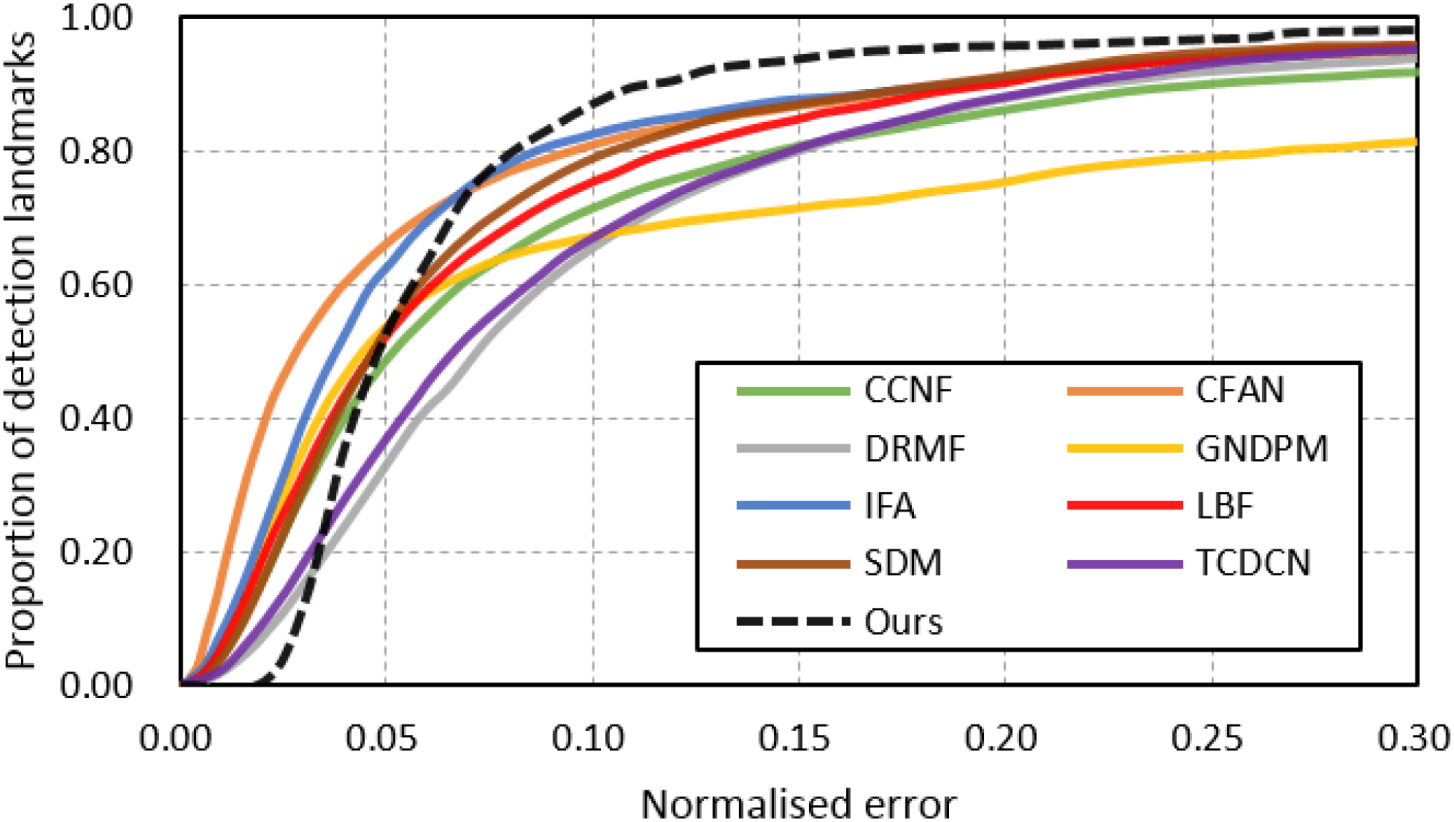
Comparison with state-of-the-art face alignment methods on the 300W dataset.

For comparison of face alignment accuracy, we implemented several state-of-the-art methods including CCNF [46], CFAN [33], DRMF [27], GNDPM [47], IFA [31], LBF [38], SDM [32], TCDCN [48], PCPR [49], CFSS [45], and Zhu *et al.* [50]. For missed faces during detection, we used prescribed face bounding boxes provided by [43] and the mean shape as the initial shape for a fair comparison. It can be observed from the CED curves in Fig 7 and the normalized error comparison results in Table 1 that our joint method outperforms most previous methods and have very similar performance with TCDCN. However, the TCDCN utilized deep neural networks and required a large amount of training data with auxiliary labels including gender, expression, appearance, etc., which limits its application to practical scenarios. Especially, benefiting from the alignment-friendly facial bounding box and the roughly aligned initial solution, our joint method achieves significant improvement over the original LBF-fast, reducing the normalized error from 7.37% to 5.55% as shown in Table 1.

**Table 1:** Results on 300W with 68-point annotation.

Method	Normalized error %
*Zhu* *et al.*	10.20
*DRMF*	9.22
*RCPR*	8.35
*SDM*	7.50
*LBF*	6.32
*CFSS*	5.76
*LBF*-*fast*	7.37
*TCDCN*	5.54
*Ours*	5.55

The improvement of face detection and face alignment by our joint method allows us to extract PPG signals based on more accurate local patches, thus resulting in a more robust performance of HR estimation especially for faces with large pose variations.

### Parameter settings

In this section, we conduct two experiments to statistically analyze the impact of the two thresholds for the skin probability and the IQR respectively and search for proper threshold values for our method. In the first experiment, we excluded the non-skin patch removal step and used patches whose IQR is within the top *n* smallest IQR among all generated local patches for PSD extraction. We set *n* to different values, from 10% to 100%, with a step of 10%. For each *n* we calculated the averaged SNR of PSDs derived from all patch pairs. Note that when *n* is set to 100%, none patches will be filtered out based on IQR. In the second experiment, we excluded the adaptive patch selection step based on IQR filtering and used patches whose averaged skin probability is below a predefined threshold *m* for PSD extraction. We set *m* to different values, from 0.0 to 0.9, with a step of 0.1. For each *m* we calculated the averaged SNR. Note that when *m* is set to 0.0, none patches will be filtered out based on skin probability.

The SNR of each PSD is calculated as in [22]. That is, SNR is calculated as the energy around the ground-truth frequency (i.e. *E*_*gt*_) plus the first harmonic of the pulse signal (i.e. *E*_1*st*−*h*_) divided by the remaining energy contained in the spectrum (i.e. *E*_*remaining*_):

$$\begin{array}{c} SNR=\frac{E_{1st-h}+E_{gt}}{E_{remaining}} \end{array}$$ (10)

**Fig 8:**
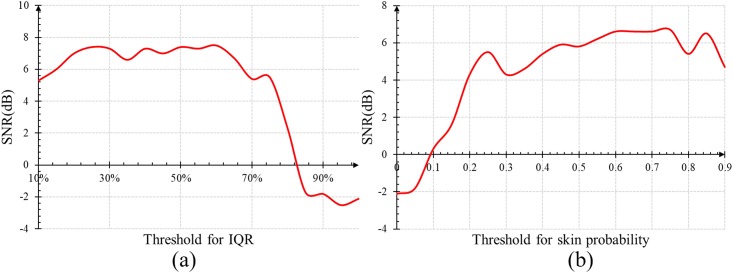
Mean SNR when varying the threshold values for (a) IQR and (b) skin probability on the validation dataset.

The two experiments were conducted on a validation dataset that is extracted from the MANNOB-HCI (Details about this public dataset are presented in Sec. Comparison of HR Measurement on MAHNOB-HCI). To avoid the overlap between the validation dataset and the test dataset used in our work, from each video we cropped a 30-second clip that is different from the 30-second clip for testing. Fig 8(a) and 8(b) show the statistical results for the first and second experiments respectively.

As can be seen in Fig 8(a), when the threshold for IQR is over 70%, the SNR of PSD drops dramatically and SNR achieves a sufficiently high and stable value when the threshold is around 50%. In Fig 8(b), the SNR of the PSD increases as the threshold for skin probability increases from 0.0 to 0.7. Further increasing the threshold to 0.9 might lead to fluctuations of SNR values. Therefore, in our experiment we chose 0.7 as the skin probability threshold and 50% as the IQR threshold for a stable and sufficiently high SNR.

### Evaluation on Pose-Variant HR dataset

#### Dataset and evaluation metrics

We constructed a Pose-Variant HR dataset by recording 20 facial videos of 10 human subjects for 30 seconds using a *SAMSUNG G9300* smartphone. The video is in 24-bit color at 30 fps with a resolution of 1920 × 1080. Each participant was asked to rotate their head 10 to 20 times with a large angle which ranges around −40° to 40° mainly in the yaw axis during video capturing. The HR groundtruth was measured using a commercial medical sphygmometer *OMRON Electronic Sphygmometer HEM-1020*. Since the sphygmometer measures HR in a 5-second window, we averaged 6 consecutive reference HR-measurements for a 30-second video to make sure that both ground truth method and our proposed method measure HR over the same window-length (i.e. 30s). We recorded 2 videos for each subject: the first video called *kineticHR* was recorded after a subject ran at a regular pace for three minutes and the second called *basalHR* was recorded when the subject was resting. The third column of Table 2 summarizes the characteristics of our Pose-Variant HR dataset.

**Table 2:** Characteristics of the two datasets used for experiments. Both datasets well emulate the realistic scenarios and are challenging for HR estimation.

Challenges	MAHNOB-HCI %	Pose-Variant HR %
*SmallPoseVariation* (‖yaw‖ < 10°)	84	100
*LargePoseVariation* (‖yaw‖ ≈ 40°)	46	50
*SmallExpressions*	100	100
*ExaggeratedExpressions*	50	0
*WithGlass*	31	50
*WithBeard*	11	0
*DarkSkin*	27	0
*PartialOcclusion*	31	0
*IlluminationChanges*	100	0

For the evaluation metrics, we used the RMSE and the Bland Altman plot. The Bland Altman plot is used to quantify the agreement between the video-based method and the contact-based method of measurements [51]. The 95% limits of agreement, estimated by mean difference ±1.96 standard deviations of the difference, provide an interval within which 95% of the differences between the measurements by the two methods. Additionally, to better demonstrate the spread of errors for each video-based method, we employed the measurement error *HR*_*error*_ = *HR*^*pred*^ − *HR*^*GT*^, where *HR*^*pred*^ and *HR*^*GT*^ are HR value predicted by video-based method and ground-truth HR, and provided the mean of *HR*_*error*_ denoted as $M_{e}=1/N\sum_{i=1}^{N}HR_{error}^{i}$, where *N* is the number of videos and $HR_{error}^{i}$ is the measurement error for the *i*^*th*^ video, and the standard deviation of measurement errors denoted as *SD*_*e*_.

#### Results

**Fig 9:**
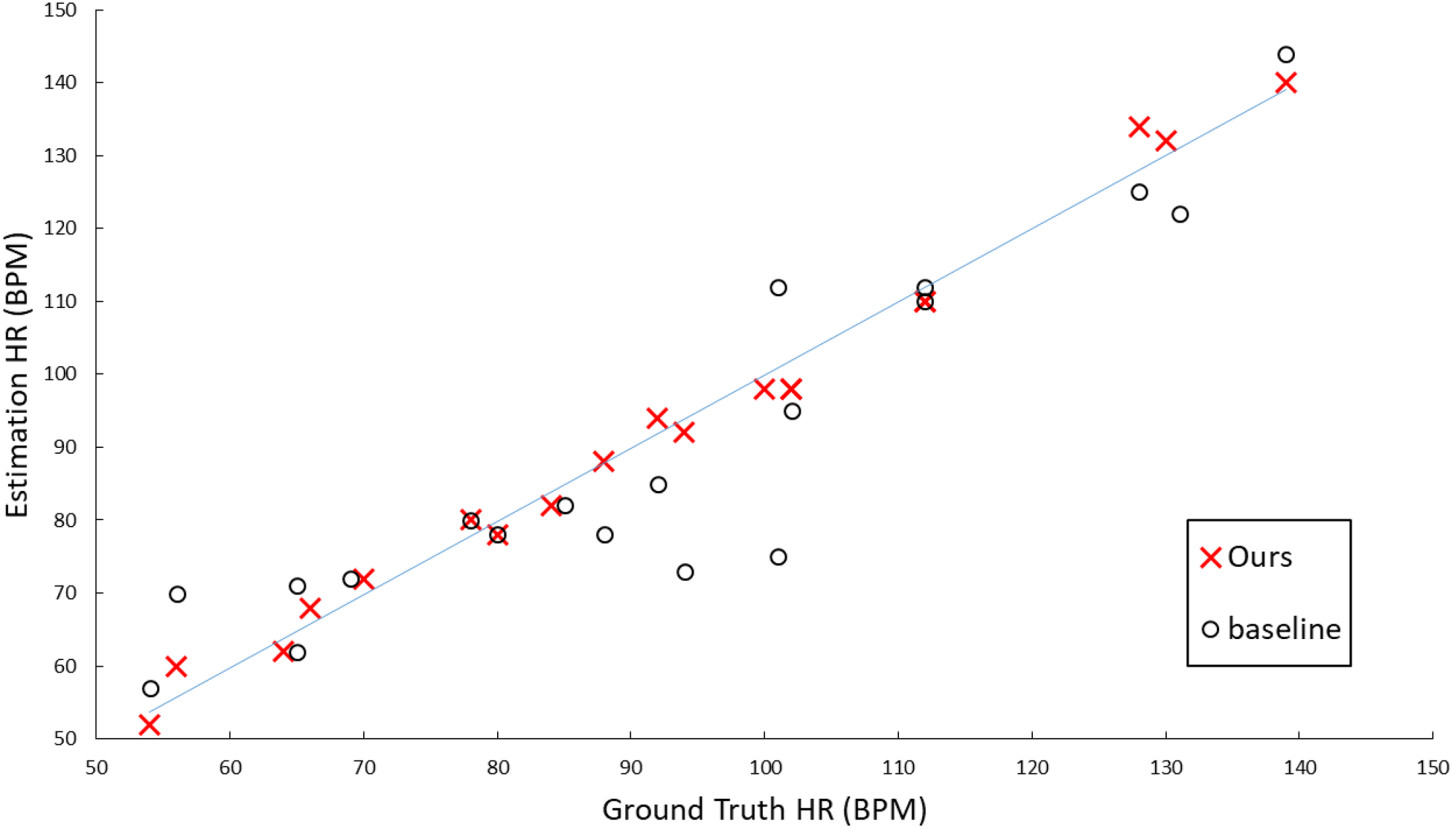
Comparison of the ground truth HR and the estimated HR by the baseline and our proposed method respectively on the Pose-variant HR dataset.

In order to evaluate the impact of our method, we built a baseline method by replacing our joint face detection and alignment method with the original LBF-fast for the first step and removing the third step from our framework in Fig 2. Fig 9 shows the comparison of the ground-truth HR and the HR estimated by baseline method and ours respectively. The Pearson correlation coefficient of the baseline is 0.86 while ours is 0.97.

**Fig 10:**
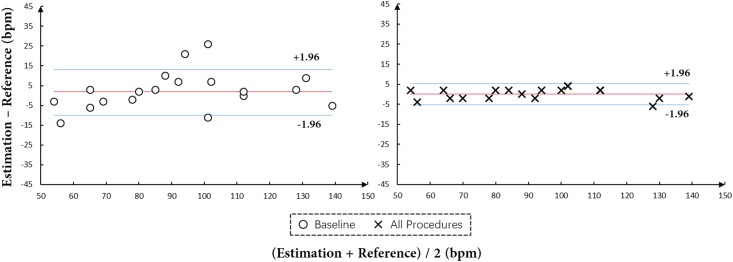
The Bland-Altman plot for HR estimates by our proposed method (right) and the baseline (left) on the Pose-Variant HR dataset.

The improvement of the two key components in the proposed algorithm was further evaluated using the Bland Altman plot, as shown in Fig 10. Fig 10 shows that almost all HR estimates by the proposed method are located inside the blue boundary lines, satisfying the 95% limits of agreement, while there are 4 out of 20 (i.e. 20%) estimates by the baseline located outside the boundary lines due to the poor facial points estimation for faces with large head motions.

To further evaluate the value of adaptive patch selection for HR estimation, we conducted an experiment in which we run all procedures except the adaptive patch selection (i.e. All Procedures w/o Adaptive Patch Selection) on the Pose-Variant dataset. Table 3 reports its RMSE, as well as those of the Baseline and the complete proposed method (i.e. All procedures). Furthermore, we performed t-test to evaluate the statistical significance of the improvements achieved by our method. Specifically, the statistical significance is denoted by the p-value, a p-value of smaller than 0.05 indicates that there is a significant difference between two sets of results achieved by different methods. As shown in Table 3, our proposed method significantly outperforms the “Baseline” method (p = 0.0295). When comparing “All Procedures w/o Adaptive Patch Selection” with “All procedures”, the results show that the adaptive patch selection reduces RMSE by 53.8% for the entire dataset (p = 0.0426), which indicates that eliminating non-skin patches and selecting patches with a stable size are indeed effective for robust HR estimation. By comparing the performance of “Baseline” with “All Procedures w/o Adaptive Patch Selection” we observe that the RMSE reduction achieved by the joint face detection and alignment method is 39.1% (p = 0.0412).

**Table 3:** RMSE (*M*_*e*_ ± *SD*_*e*_) of three methods: Baseline, ours with all procedures except adaptive patch selection, and ours with all procedures.

Method	basalHR	kineticHR	Overall
*Baseline*	8.7 (0.8±8.7)	10.3 (4.6±9.9)	9.2(2.6±9.5)
*All Procedures w/o Adaptive Patch Selection*	5.3 (1.4±5.1)	5.2 (−0.3±5.2)	5.2 (0.6±5.2)
*All procedures*	2.1 (−0.2±2.3)	3.2 (0.6±3.0)	2.4(0.2±2.7)

### Comparison of HR measurement on MAHNOB-HCI

#### Dataset and evaluation metrics

**Fig 11:**
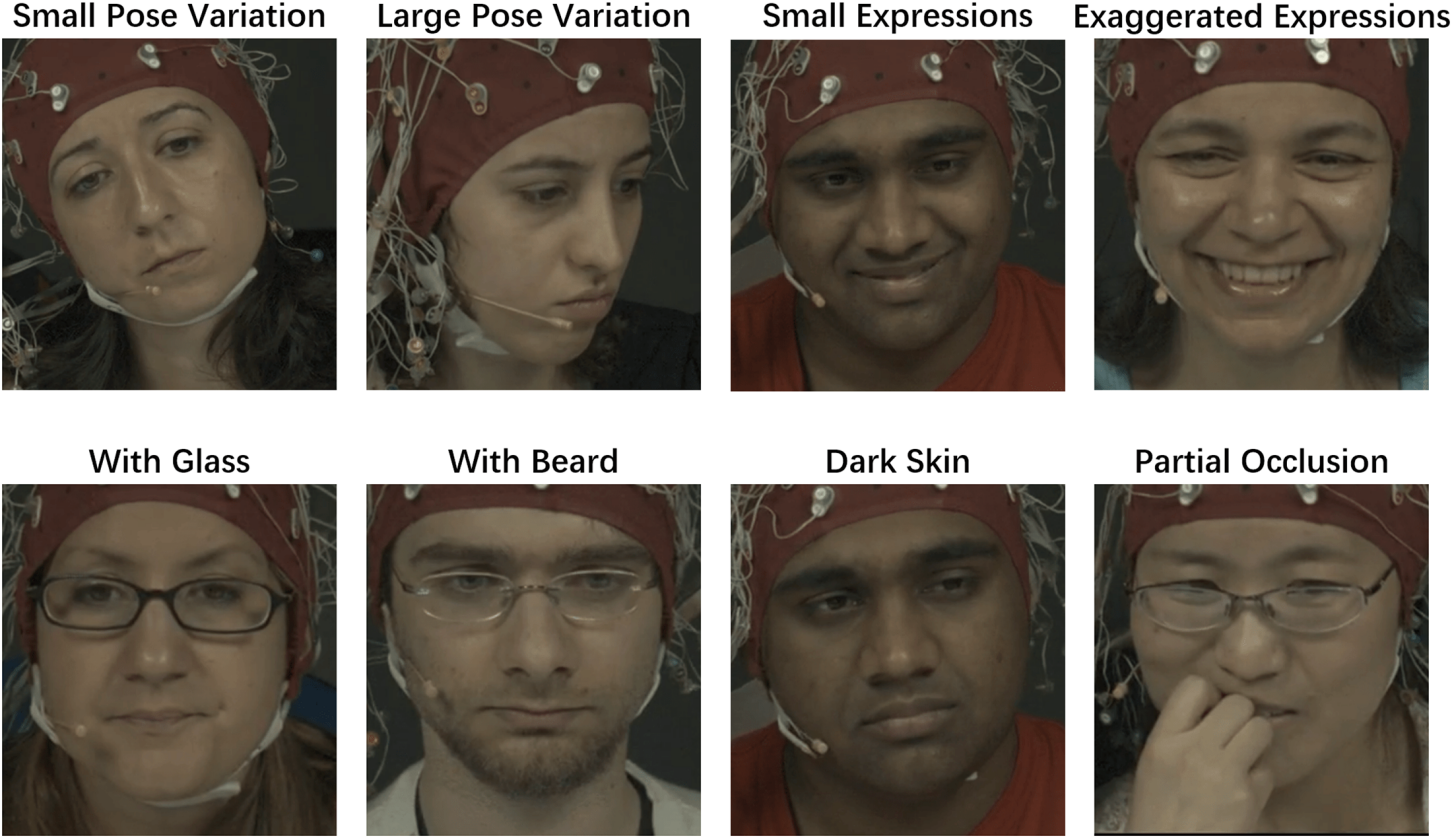
Examples of challenging cases in the MAHNOB-HCI dataset. Since participants were facing a flashing screen when the videos were being captured, the challenge of illumination changing are present in all videos.

Although on the self-collected Pose-Variant HR dataset our method shows a good performance of HR estimation and a robustness to a large head pose variance, we still want to demonstrate our method by answering two questions: 1) whether our method is consistently robust on a more comprehensive dataset which contains various challenges other than large head pose variance for the HR estimation (e.g. different skin tones, illumination changes and facial expressions), well simulating realistic scenarios, and 2) whether our method is consistently robust on a larger dataset which contains more than hundreds of video clips. To answer these two questions, we evaluated our method on a public dataset MAHNOB-HCI [36]. This dataset contains 600 facial videos captured in 24-bit color at 61 frames per second with a resolution of 780 × 580 from 30 human subjects. ECG signals were recorded at 256 Hz using three sensors attached to each participant’s body, which allows precise identification of heart beats and consequently to compute the ground truth HR. For a fair comparison, we used the same video configuration as [1], i.e. choosing the first 27 human subjects, which provides totally 487 facial videos, a 30-second clip from each video, from frames 306 to 2,135, is used for our test. The groundtruth HR is calculated via a QRS-detection algorithm [52] from the portion of the corresponding ECG of the second channel (EXG2, upper left corner of the chest). The MAHNOB-HCI dataset presents various challenges for the HR estimation including large head motions, dynamic illumination changes, various skin tones and partial occlusions. The second column of Table 2 summarizes the characteristics of the dataset and examples of some each challenging cases are shown in Fig 11.

To well demonstrate the robustness to different challenges of our method, we re-organized the MAHNOB-HCI and created 8 subsets by sampling from the MAHNOB-HCI dataset. Each subset consists of 20 video clips. According to the most dominated challenge in each subset, these 8 subsets are called respectively *Stationary*, *Large Pose*, *Expression*, *With glass*, *With beard*, *White*, *Light brown* and *Dark brown/black*. For the *Stationary* subset, video clips are collected from challenges cases of Small Pose Variation and Small Expressions in the Table 2. For the *Large Pose* subset, video clips mainly contain large pose variation, where heads rotate from −40° to 40° mainly in the yaw axis. Since it is quite difficult to quantitatively measure facial expressions, the *Expression* subset is constructed from subjectively selected video clips in which the subjects laugh (as shown in Fig 11), frown or are astonished. We believe such 8 subsets can well cover almost all possibly occurred challenges in realistic scenarios.

We first compared three methods: Baseline, All Procedures w/o Adaptive Patch Selection and All procedures on the 8 subsets using the Bland Altman plot to understand the impact of each module in our method on addressing the 3 challenges (i.e. different motions, occlusions, and different skin tones). Then we compared the overall performance of HR estimation based on our method vs. three state-of-the-art methods on the entire 487 videos from the MAHNOB-HCI dataset using RMSE and the well-estimation rate, i.e. the percentage of testing samples whose absolute errors are less than 5 bpm [1], which are widely used evaluation metrics for HR measurement.

#### Results

**Fig 12:**
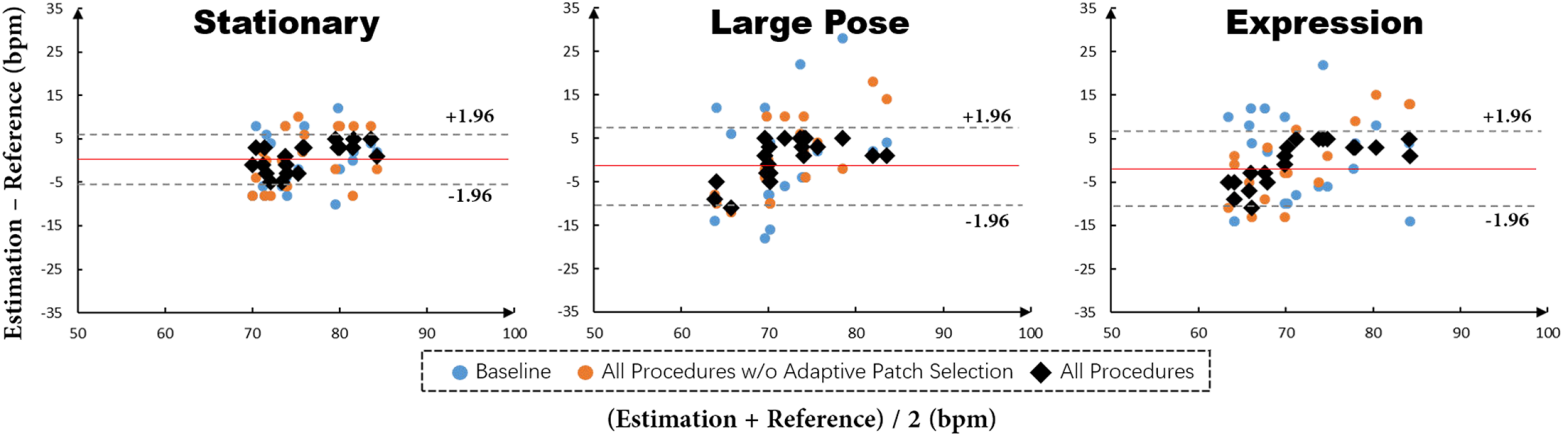
Evaluations results of baseline, all procedures w/o adaptive patch selection and all procedures on the subsets of *Stationary*, *Large Pose* and *Expression*. The red line in each plot indicates the mean value of differences between All procedures and the reference. The pair of dashed lines in each plot is mean±1.96*σ* between All procedures and the reference to denote the variance range.

Fig 12 illustrates the impact of joint face detection and alignment, and adaptive patch selection on addressing problems induced by motions: 1) tracking-artifacts resulted from head motions (i.e. translation, scaling and yaw rotation), and 2) facial expressions. Reading plots from left to right we can observe that both challenges of large pose variance and facial expression significantly degrade the performance of Baseline. Using joint face detection and alignment, i.e. All Procedures w/o Adaptive Patch Selection, can improve the performance. Further integrating the adaptive patch selection into the method can provide non-exclusive improvements. As shown in Fig 12, black dots corresponding to All procedures cluster tightly, which indicates that more accurate facial landmarks and adaptive patch selection strategy do greatly improve the SNR and enable our method robust to both motion-artifacts (including translation and rotating) and expression-artifacts.

**Fig 13:**
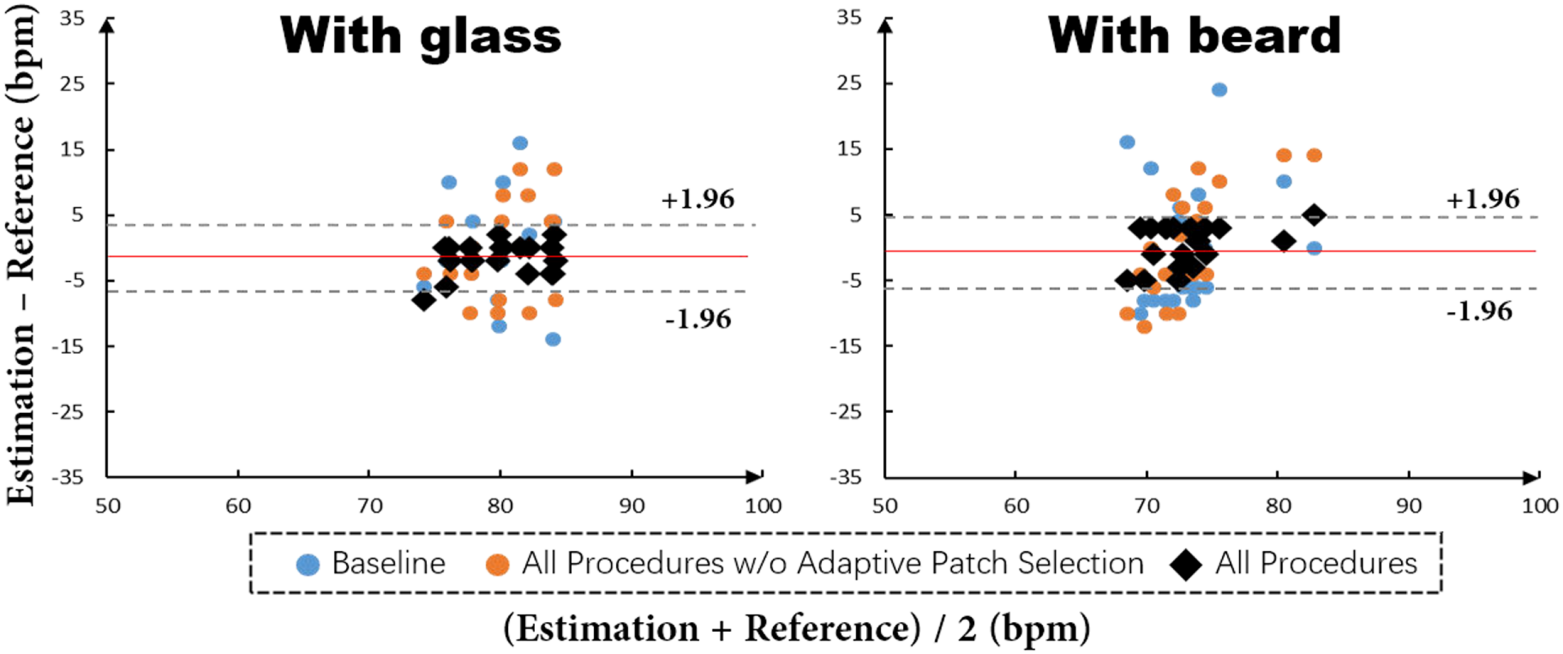
Evaluations results of baseline, all procedures w/o adaptive patch selection and all procedures on the subsets of *With glass* and *With beard*. The red line in each plot indicates the mean value of differences between All procedures and the reference. The pair of dashed lines in each plot is mean±1.96*σ* between All procedures and the reference to denote the variance range.

To demonstrate the robustness of our method to occlusions mainly due to glasses and beards, we evaluated the three methods on the subsets of *With glass* and *With beard*. Comparison results in Fig 13 show that the proposed adaptive patch selection provides obvious performance improvements when comparing black dots with orange and blue dots. The performance gain is mainly achieved by excluding patches with few skin pixels which could decrease the SNR and in turn pose difficulties for HR estimation. In contrast, our proposed SPM model in the adaptive patch selection strategy can greatly remove such patches, improving the final performance.

**Fig 14:**
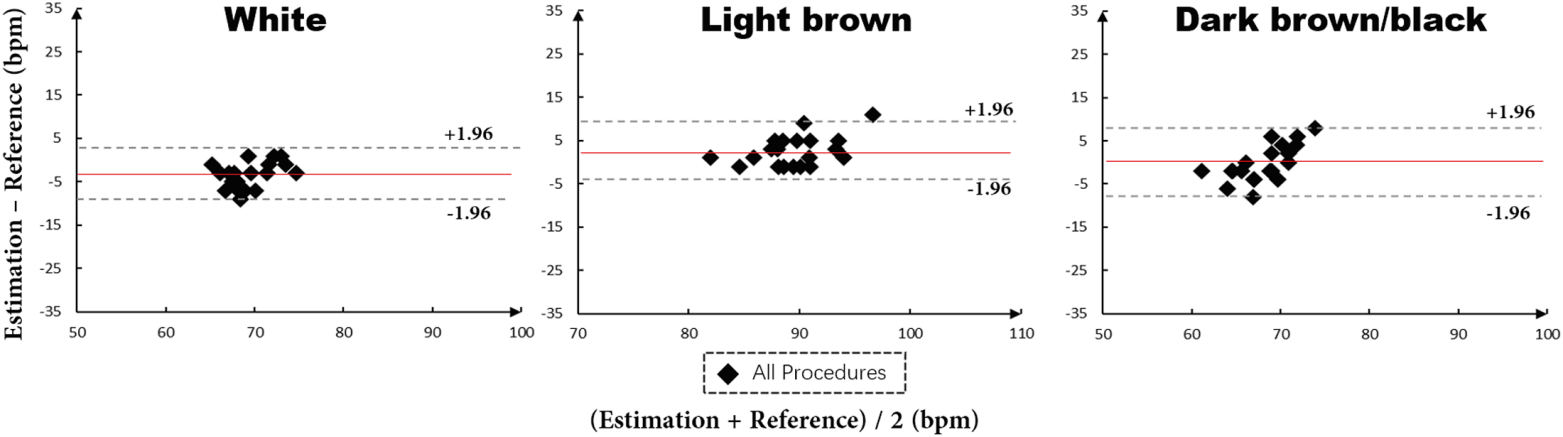
Evaluations results of all procedures on the subsets of *White*, *Light brown* and *Dark brown/black*. The red line in each plot indicates the mean value of differences between All procedures and the reference. The pair of dashed lines in each plot is mean±1.96*σ* between All procedures and the reference to denote the variance range.

For different skin tones, we evaluated only the All procedures on the subsets of *White*, *Light brown* and *Dark brown/black* since it is hard to find video samples from the MAHNOB-HCI dataset that only have skin tone variance without head moving and rotating and facial expression or occlusions. Moreover, experimental experience indicated that there is a more significant impact on HR estimation by head pose variance and occlusions than skin tone variance. The three plots in Fig 14 show that there is a high agreement between our method and the reference for all the three kinds of different skin tones.

It should be noted that the robustness of our method to illumination changes, which is an important issue in realistic scenarios, has been already demonstrated among all experiments above, since all participants were facing a flashing screen when the videos were captured. To summarize, for more various challenges under realistic scenarios including large pose variants, facial expressions, partial occlusions and different skin tones, our method demonstrates a consistently robust performance benefiting from more reliable pulse signals extracted by the proposed joint face detection and alignment and adaptive patch selection strategy.

To answer the question that whether our method is consistently robust on a larger dataset which contains more than hundreds of video clips, we evaluated our method on all 487 video clips from the MAHNOB-HCI dataset and such amount of videos is ∼30 times larger than the self-collected Pose-Variant HR dataset. Moreover, we compared our method with three state-of-the-art HR estimation methods including a face detection-based HR estimation method, i.e. Poh2011 [3], and two face alignment-based methods, i.e. Li2014 [2] and Lam2015 [1]. Li2014 and Lam2015 adopted a similar idea that tracks a fixed local facial patch around the nose and the mouth based on face alignment for HR estimation. Table 4 shows the experimental results.

**Table 4:** Results of HR estimation on the MAHNOB-HCI (best performance in bold).

Method	RMSE (*M*_*e*_±*SD*_*e*_)	Well-estimation rate %
*Poh*2011	21.3	46.2
*Li*2014	15.0	68.1
*Lam*2015	8.9	75.1
*Baseline*	17.4	61.0
*All Procedures w/o Adaptive Patch Selection*	11.3 (2.5±11.5)	71.0
*Ours* (*All Procedures*)	**7.4** (1.4±7.1)	**79.2**

It is not surprising that the face alignment-based methods (Li2014 and Lam2015) achieve better performance than the face detection-based method (Poh2011), since non-skin pixels, which have negative effects on PPG extraction, are excluded for some cases through selection of specific local facial region based on landmarks produced by face alignment. For the two face alignment-based methods, Li2014 only used a single predefined irregular shaped mask which may fail to estimate the HR when there are many noisy sources in the mask (e.g. beards), while Lam2015 extracted PPG signals from multiple local patches and combined their HR estimates by a majority voting scheme for the final HR, increasing the robustness of HR estimation.

Compared with Lam2015 [1], it is much easier for FastICA to identify a correct PPG signal in our method based on three reasons: 1) local patches are generated based on more accurate facial landmarks, minimizing the adverse impact of tracking-artifacts, 2) non-skin patches including eyes, glass and beard-contained regions are excluded and it is incapable to recovering correct signals from such patches, and 3) unreliable patches are rejected by selecting only size-stable patches on the timeline, providing raw signals with relatively high SNR for robust HR estimation. More specifically, head motion/facial expression could induce non-trivial amount of missing pixels in local patches and in turn make the two coefficients in Eq (9) be time-varying variables, causing inaccuracy in solving Eq (9) using ICA. To summarize, our method can remove as many outliers (i.e. local patches producing low-SNR raw signals) as possible and hence quadratically improve the probability of selecting a pair of inlier patches for deriving a correct PPG signal using FastICA. Accordingly, even though considering the measurement errors and resolution of ECG signals, RMSE of 8.9 bpm achieved by Lam2015 was considered as a result close to saturation, our method still achieves another 12% reduction of RMSE, which demonstrates the effectiveness of our joint detection and alignment method for handling large pose variations and adaptive patch selection for discovering useful patches.

It is noteworthy that there is a recent work by Tulyakov *et. al.* [20] also achieved the state-of-the-art performance on the MAHNOB-HCI dataset, but we did not compare our method directly with theirs mainly due to two reasons. First, they adopted an old video configuration of the MAHNOB-HCI dataset which contains 527 videos and we can no longer reproduce such configuration since the MAHNOB-HCI dataset has been updated from time to time. Second, there is no released source code and it is quite difficult to re-implement their work due to the lack of implementation details and parameter configurations of their method. However, we can still provide an indirect comparison between our method and [20]. We believe that the new video configuration used in our work is more challenging than the old one, since Poh2011 [3] and Li2014 [2] evaluated their methods on MAHNOB-HCI of both old and new configurations. The reported results in [1] show that the performance of Poh2011 [3] and Li2014 [2] on the old configuration is much better than those achieved on the new configuration (RMSE of 13.6 bpm and 7.62 bpm respectively on the old one and RMSE of 21.3 bpm and 15.0 bpm respectively on the new configuration). Despite of more challenging cases in our configuration, our method still provides performance close RMSE value to [20], whose RMSE on the old and easier video configuration is 6.32 bpm.

**Fig 15:**
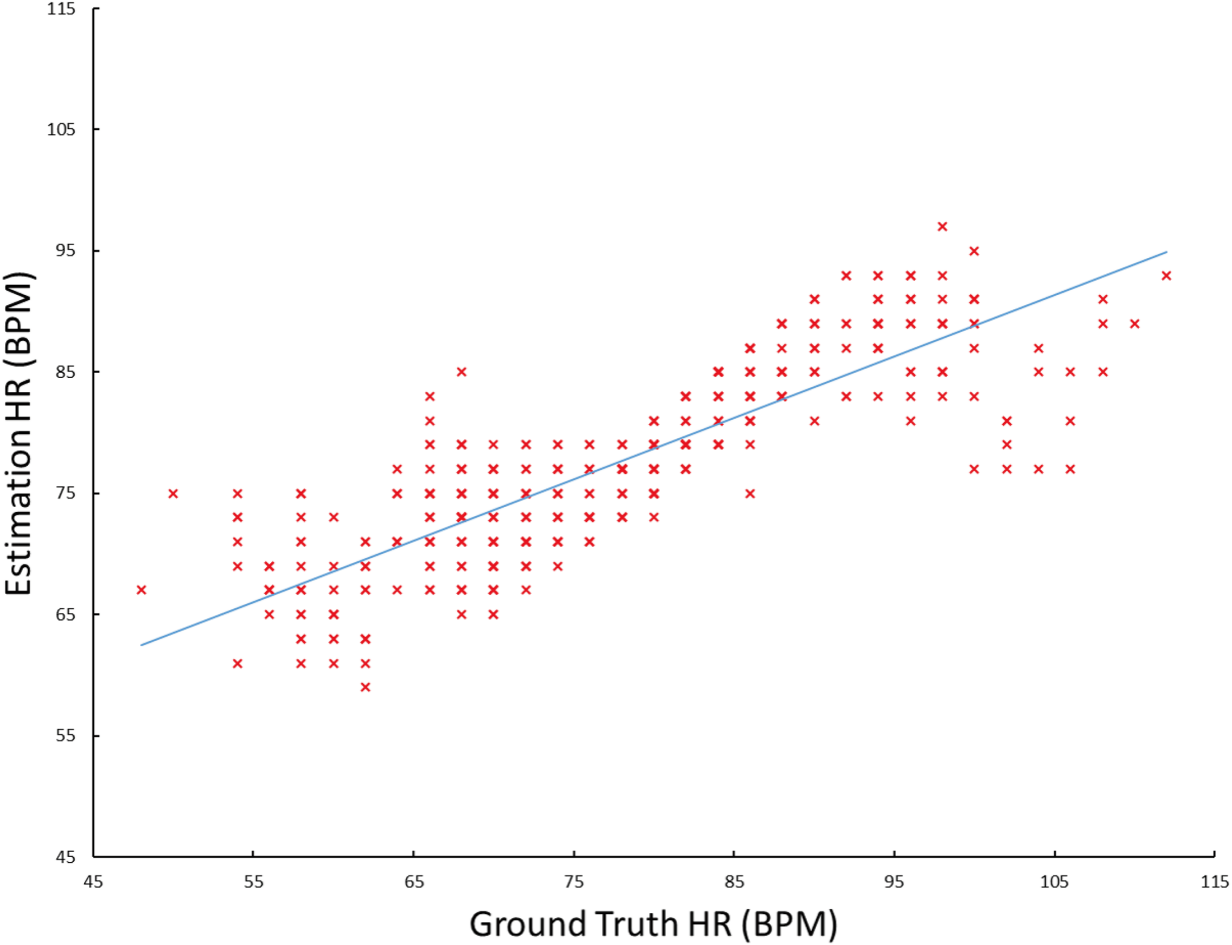
Comparison of the ground truth HR derived from ECG and the estimated HR by our proposed method on MAHNOB-HCI.

Fig 15 shows the comparison results of the HR measured by our method and the ground truth HR based on the MAHNOB-HCI dataset with new video configuration. The Pearson correlation coefficient is 0.84, which means that overall HR estimation is well correlated with the ground-truth. Moreover, we checked the outliers in our HR estimation and found that the failure usually occurs when the participants’ faces are largely occluded by hands or even out of the screen.

## Conclusion and future work

In this study, we present a HR estimation method that can robustly and accurately measure the human HR from a facial video under large head motions, facial expressions, partial face occlusions or dynamic illuminations. First, our method employs a joint face detection and alignment method by incorporating a well-designed multipose face detector with facial alignment initialization in a unified cascaded framework. The joint process could ensure a fast and accurate convergence of face alignment. Comparing to the state-of-the-art alignment methods, the proposed joint method achieves 25% accuracy improvement, and in turn greatly reduces the motion artifacts for HR estimation. Second, a DT algorithm is applied to generate triangular non-overlapping local patches by connecting localized landmarks, reducing the negative effects of non-rigid motions. Then a SPM is constructed to filter out non-skin patches and the remaining patches whose sizes remain stable across the temporal axis are adaptively selected for extracting PPG signals. Experimental results on a self-collected dataset Pose-Variant HR and a larger and comprehensive dataset MAHNOB-HCI consistently demonstrated our method’s superior performance to the state-of-the-art methods under realistic scenarios.

In addition to estimating an average HR for a given video, our method can also extract other vital signals with a few minor modifications. For example, we can average all raw signals that vote the final estimated HR, and obtain the pulse signal by filtering the averaged signal using a band-pass filter. The pulse signal can deliver more information besides HR, e.g. heart rate variability (HRV). We will further explore such idea in our future work.
